# Circuits Regulating Pleasure and Happiness in Bipolar Disorder

**DOI:** 10.3389/fncir.2017.00035

**Published:** 2017-05-22

**Authors:** Anton J. M. Loonen, Ralph W. Kupka, Svetlana A. Ivanova

**Affiliations:** ^1^Groningen Research Institute of Pharmacy, University of GroningenGroningen, Netherlands; ^2^GGZ WNB, Mental Health HospitalBergen op Zoom, Netherlands; ^3^Department of Psychiatry, VU University Medical CenterAmsterdam, Netherlands; ^4^Tomsk National Research Medical Center of the Russian Academy of Sciences, Mental Health Research InstituteTomsk, Russia; ^5^Department of Ecology and Basic Safety, National Research Tomsk Polytechnic UniversityTomsk, Russia

**Keywords:** bipolar disorder, stress, mechanism, basal ganglia, amygdala, habenula, neuroplasticity

## Abstract

According to our model, the motivation for appetitive-searching vs. distress-avoiding behaviors is regulated by two parallel cortico-striato-thalamo-cortical (CSTC) re-entry circuits that include the core and the shell parts of the nucleus accumbens, respectively. An entire series of basal ganglia, running from the caudate nucleus on one side to the centromedial amygdala on the other side, control the intensity of these reward-seeking and *misery*-fleeing behaviors by stimulating the activity of the (pre)frontal and limbic cortices. Hyperactive motivation to display behavior that potentially results in reward induces feelings of hankering (relief leads to pleasure); while, hyperactive motivation to exhibit behavior related to avoidance of aversive states results in dysphoria (relief leads to happiness). These two systems collaborate in a reciprocal fashion. We hypothesized that the mechanism inducing the switch from bipolar depression to mania is the most essential characteristic of bipolar disorder. This switch is attributed to a dysfunction of the lateral habenula, which regulates the activity of midbrain centers, including the dopaminergic ventral tegmental area (VTA). From an evolutionary perspective, the activity of the lateral habenula should be regulated by the human homolog of the habenula-projecting globus pallidus, which in turn might be directed by the amygdaloid complex and the phylogenetically old part of the limbic cortex. In bipolar disorder, it is possible that the system regulating the activity of this reward-driven behavior is damaged or the interaction between the medial and lateral habenula may be dysfunctional. This may lead to an adverse coupling between the activities of the *misery*-fleeing and reward-seeking circuits, which results in independently varying activities.

## Introduction

An essential characteristic of bipolar disorder is patients experience episodes of mania (or, if less severe, hypomania, which will be considered as essentially the same throughout this paper) and usually also episodes of depression (American Psychiatric Association, 2013). The lifetime prevalence of bipolar disorder is far less than that of major depressive disorder (MDD; American Psychiatric Association, 2013). In the NEMESIS-2 study of 18–65 year-old Dutch citizens, the lifetime prevalence for bipolar disorder, according to criteria of the Diagnostic and Statistical Manual of Mental Disorders, Fourth Edition, Text Revision (DSM-IV-TR; American Psychiatric Association, 2000), was 1.2% in men and 1.4% in women; while, it was 13.1% and 24.3%, respectively, for MDD (De Graaf et al., 2010). Moreover, bipolar disorder usually starts in adolescence or young adulthood; while, many women suffer from their first depressive episode after menopause or even in their senium. Therefore, for women, the risk of depression once in their lifetime may be as high as 40%. Perhaps, the majority of clinical depression episodes are the most suitable reaction to unbearable stressful circumstances that exhaust the individual (Loonen and Ivanova, 2016a). In this context, part of MDDs may be considered a reaction, like fever, vomiting, or diarrhea, which can improve one's chances of staying alive under difficult circumstances. However, in bipolar disorder, the situation may be entirely different. Here, the mechanisms to react to the normal challenges of daily living, may be dysfunctional. This dysfunction results in increased or decreased goal-directed behavior aimed at improvement of the individual's circumstances of living.

Almost three decades ago, Richard Depue and colleagues started to develop a “behavioral approach system (BAS) dysregulation theory” of bipolar spectrum disorders (Urošević et al., 2008; Nusslock et al., 2009; Bender and Alloy, 2011). Briefly, this theory holds that individuals with bipolar disorder experience extreme fluctuations in activation and deactivation of the BAS, which is involved in approach to reward, and these fluctuations are reflected in bipolar symptoms. This corresponds to the psychobiological system of Jeffrey Gray (Gray, 1981), who also described a behavioral inhibition system (BIS) regulating inhibition of behavior in response to threat and punishment (Nusslock et al., 2009). Recently, a new model was presented that distinguished between two types of cortico-ganglio-thalamo-cortical tracks, and could regulate intuitive (emotional) and skilled (cognitive) behaviors. These tracks were called the limbic and extrapyramidal circuits, respectively (Loonen and Ivanova, 2015). The motivation for these two types of behaviors was proposed to be regulated by two re-entry circuits including the nucleus accumbens shell (NAcbS) and the nucleus accumbens core (NAcbC), respectively. The NAcbS-containing circuit motivated a display of *misery*-fleeing behavior; while, the NAcbC-containing circuit motivated reward-seeking conduct. *Misery* refers here to experiencing all types of aversive circumstances like danger, heat, cold, loss, or pain, hence not only “misery” in an anthropomorphic sense.

**Figure 1:**
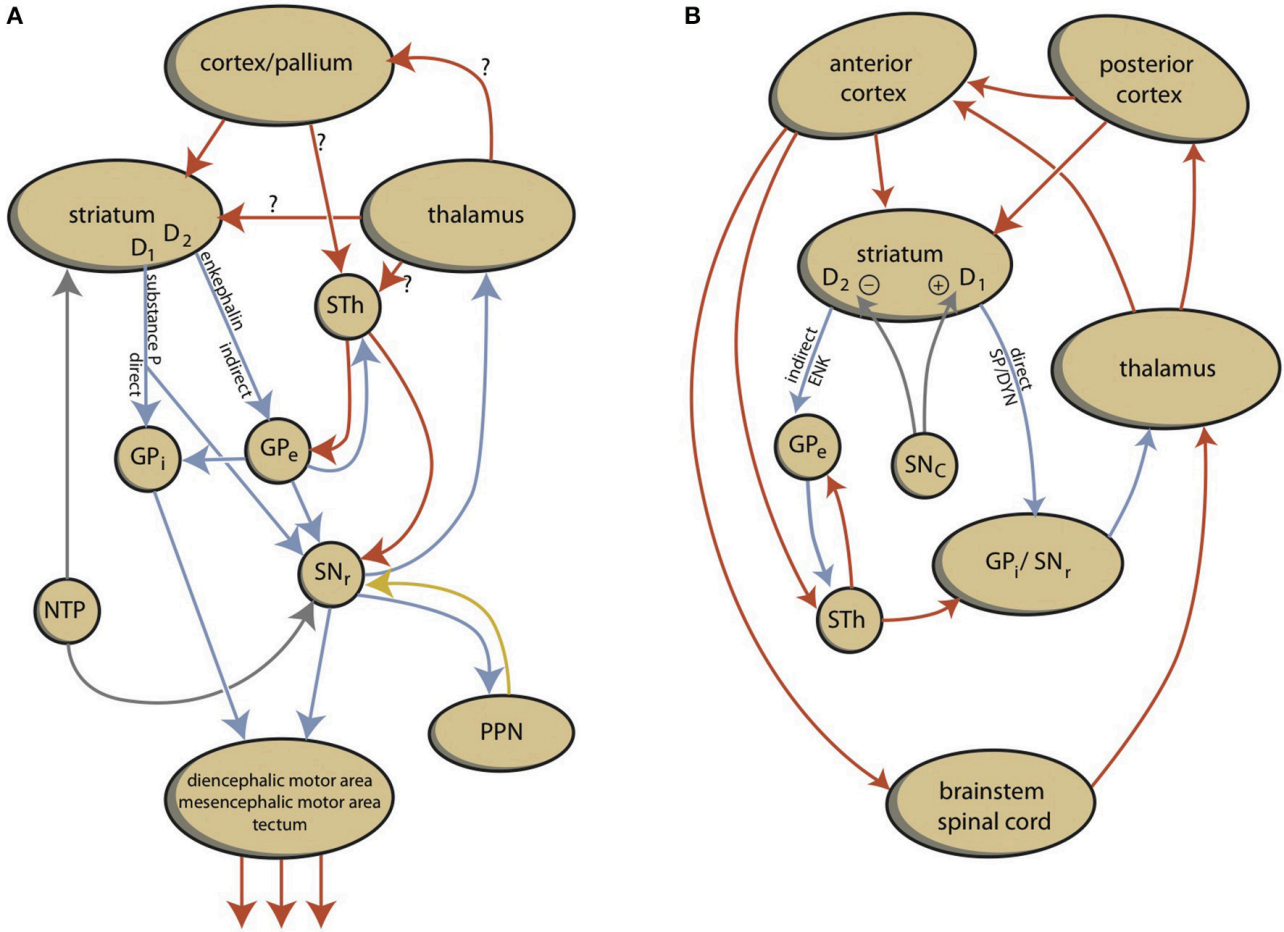
**Schemes representing the organization of lamprey (A)** and human **(B)** extrapyramidal systems. D1, dopamine D1 receptor type carrying medium spiny neurons; D2, dopamine D2 receptor type carrying medium spiny neurons; DYN, dynorphin; ENK, enkephalin; GPe, external segment of globus pallidus; GPi, internal segment of globus pallidus; NTP, nucleus of the tuberculum posterior; SP, substance P; PPN, pedunculopontine nucleus; SNc, substantia nigra pars compacta; SNr, substantia nigra pars reticulata; STh, subthalamic nucleus.

A basic starting point of this theory is that, in its most essential form, behavior can be considered an adaptive reaction of organisms to important stimuli from the environment. To continue living as an individual and a species, even our oldest ancestors living in the oceans over 540 million years ago must have been capable of reacting to the environment to feed, defend, or reproduce. So, their primitive nervous systems must have been capable of regulating such necessary behavior, and must have contained the most essential structures of all of today's Animalia. Therefore, we reviewed the literature concerning the evolution of the forebrain in vertebrates (Loonen and Ivanova, 2015). A creature comparable to today's lamprey was the earliest vertebrate with a modern forebrain. It was concluded, that essential parts of the regulatory mechanisms of lampreys may be conserved in humans. Lampreys have a separate dorsal pallium, which preceded the newest part of the cerebral cortex in more recent vertebrates; however, it does not function as in humans. In addition, they possess a quite modern extrapyramidal system; although, it is not yet a cortical-subcortical circuit including the dorsal pallium. Motor activity is regulated directly by the extrapyramidal system, which controls diencephalic and mesencephalic motor centers (Figure 1). It has been proposed that the striatum of lampreys corresponds to the ganglionic part of the amygdaloid complex of humans. In addition, lampreys have a well-developed habenula and this system regulates the activity of reward-bringing or *misery*-avoiding behavior. The activity of the lateral part of the habenula (LHb) is regulated by a specific structure called the “habenula-projecting globus pallidus” (GPh).

In the current paper, we propose that in bipolar disorder, two pathological mechanisms mutually contribute to generating the pathological mood episodes. The first mechanism is comparable to the mechanism inducing major depressive episodes in MDD. This is primarily related to pathological hyperactivity of the *misery*-fleeing system, which results in hypoactivity of the reward-seeking system (Loonen and Ivanova, 2016a). However, in bipolar disorder, a pathological mechanism induces the reward-seeking system to react with either hypoactivity or hyperactivity. In addition, the hyperactive reward-seeking system may not always induce the *misery*-fleeing system to switch the mood to euphoria. The combination of these processes can result in anhedonic depression, agitated depression, mixed depressive state, mixed manic state, dysphoric mania, and euphoric mania. Regulating the activity of the reward-seeking system is an important function of the habenula. We will summarize the biological findings to confirm or refute this hypothesis with specific reference to the role of the habenula.

## Description of our model

Behavior can be considered a mechanism where the brain manages input to create a specific output that enables the organism to adapt to the ever changing circumstances within its biosphere. In humans, input from the senses is primarily translated within the cerebral cortex into a specific behavioral output. Sensory information is processed within the posterior cerebral cortex in a stepwise fashion (Loonen et al., 2016). Specific information is integrated with other sensory information and transmitted from the primary sensory cortex to the secondary sensory cortex, then to the association cortex, and so on. Within the anterior cerebral cortex, a similar diverging flow of information occurs that leads to the activation of specific brain regions, e.g., the motor cortex. Apart from this stepwise analysis, other fibers connect to more distant regions that run in parallel. Every neural connection is capable of learning, due to the characteristics of glutamatergic transmission, which can increase or decrease the sensitivity of connecting synapses by inducing long term potentiation (LTP) or long term depression (LTD). Therefore, the cortex can “learn” to transmit specific sensory information to a specific output unit via a “preferred” cortical tract. Accordingly, the cerebral cortex learns to interpret sensory information and to produce a specific behavioral response.

**Figure 2:**
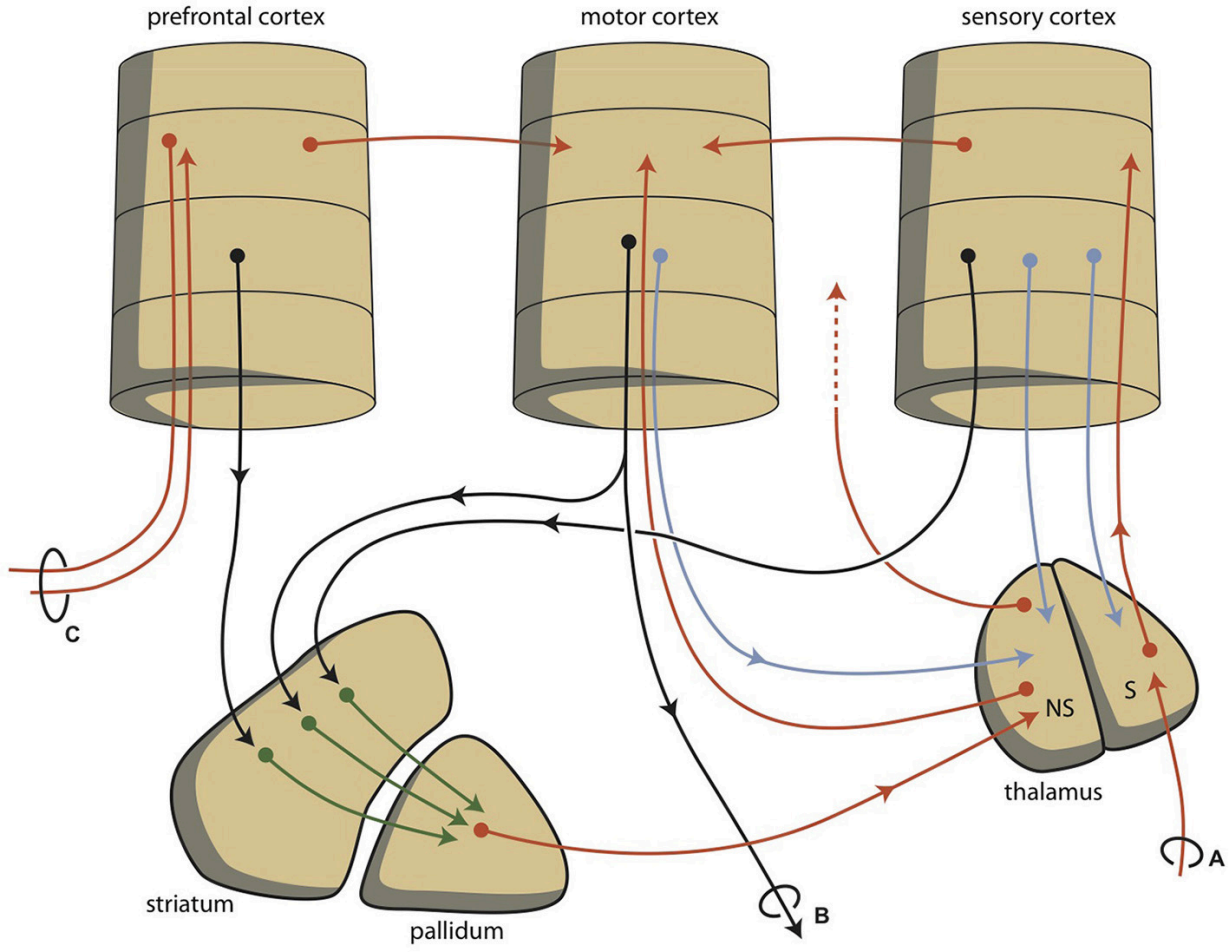
**Schematic representations of converging extrapyramidal circuits. (A)** sensory input; **(B)** motor output; **(C)** contralateral communication; NS, non-specific; S, specific.

Although this process is expedient, it can be highly sensitive to dysregulation, both in routine functions and learning. Therefore, a parallel circuit has evolved, which includes subcortical structures (Figure 2). All processing units in the cerebral cortex also send information to the basal ganglia (Heimer, 2003). The route through the basal ganglia and thalamus targets corresponding processing units in the anterior cortex (Loonen and Ivanova, 2013). This parallel circuit has stimulatory and inhibitory pathways and its glutamatergic synapses can also induce LTP and LTD (Figure 1). Therefore, this parallel route through the basal ganglia enables the brain to correct serially transmitted information, when it arrives at the “final” destination. Moreover, the connection through the basal ganglia is convergent (Figure 2; Alexander et al., 1986; Loonen, 2013; Loonen and Ivanova, 2013). Hence, the processing units in the posterior and anterior cortices and their outputs converge within this subcortical circuit and have the same frontal cortical output unit. Again, the “learning” ability of glutamatergic synapses within this framework make it possible to process a constantly varying input and produce very complex, sophisticated output patterns in a reproducible, precise fashion.

This organization of connections is well-known as the extrapyramidal system, which regulates cognition and movements (Groenewegen, 2003). In our depression model, we suggest that a similar organization can be distinguished within the limbic cortex; although here, the structure is more complex and less modular due to the ancient origins of these structures (Loonen and Ivanova, 2015, 2016a,b; Loonen et al., 2016). To simplify, we have concentrated on the primary limbic cortex within the superficial and deep corticoid regions of the amygdala. These cortical regions are connected with many other archicortical and mesocortical areas. The corticoid amygdalar regions can be considered input areas, and the centromedial (ganglionic or nuclear) region can be considered the output area of the amygdaloid complex. The stria terminalis connects this nuclear amygdala to amygdaloid pallidum (i.e., the bed nucleus of the stria terminalis) and to the diencephalon, and from these structures, the anterior dorsomedial frontal areas can be reached. However, the majority of output from the limbic basal ganglia flows to the brainstem. This connection is probably related to the absence of cortical motor centers in our earliest vertebrate ancestors (Loonen and Ivanova, 2015). In lampreys, movements are directed by diencephalic and brainstem motor centers (Loonen and Ivanova, 2015). In our opinion, in higher vertebrates, the nuclear amygdala represents the striatum of our oldest vertebrate ancestors. Nevertheless, the amygdala affects the motor output of higher vertebrates, including humans, by inducing the drive to seek food, warmth, comfort, etc., or to escape from pain, thirst, discomfort, etc. (Sewards and Sewards, 2003).

Hence, two types of cortical-subcortical circuits may be distinguished: extrapyramidal and limbic. These systems have different first step relay stations. The extrapyramidal circuit includes the caudate nucleus, putamen, and nucleus accumbens core (NAcbC). The limbic circuit includes the extended amygdala (consisting of nuclear amygdala, connecting extended amygdala, bed nucleus of the stria terminalis), and the nucleus accumbens shell (NAcbS). The extrapyramidal circuit regulates “rational,” cognitively constructed, skilled behavior, which is often goal-oriented and includes decision making. The limbic circuit regulates emotional (instinctive and automatic) behaviors, which are often defensive, and this regulation includes (attentive) salience. The two systems influence each other in a reciprocal (yin-and-yang-like) fashion; moreover, both systems can inhibit or activate, depending on the situation. It is generally accepted that the prefrontal cortex (PFC) selects the appropriate response (Stuss and Knight, 2002; Fuster, 2008). The dorsolateral PFC is particularly important for controlling rational responses, and the medial PFC controls emotional responses. Within the medial PFC, the orbitofrontal cortex (OFC) plays a particularly noteworthy role because it is essential for regulating the direction of motivation (Zald and Rauch, 2006).

**Figure 3:**
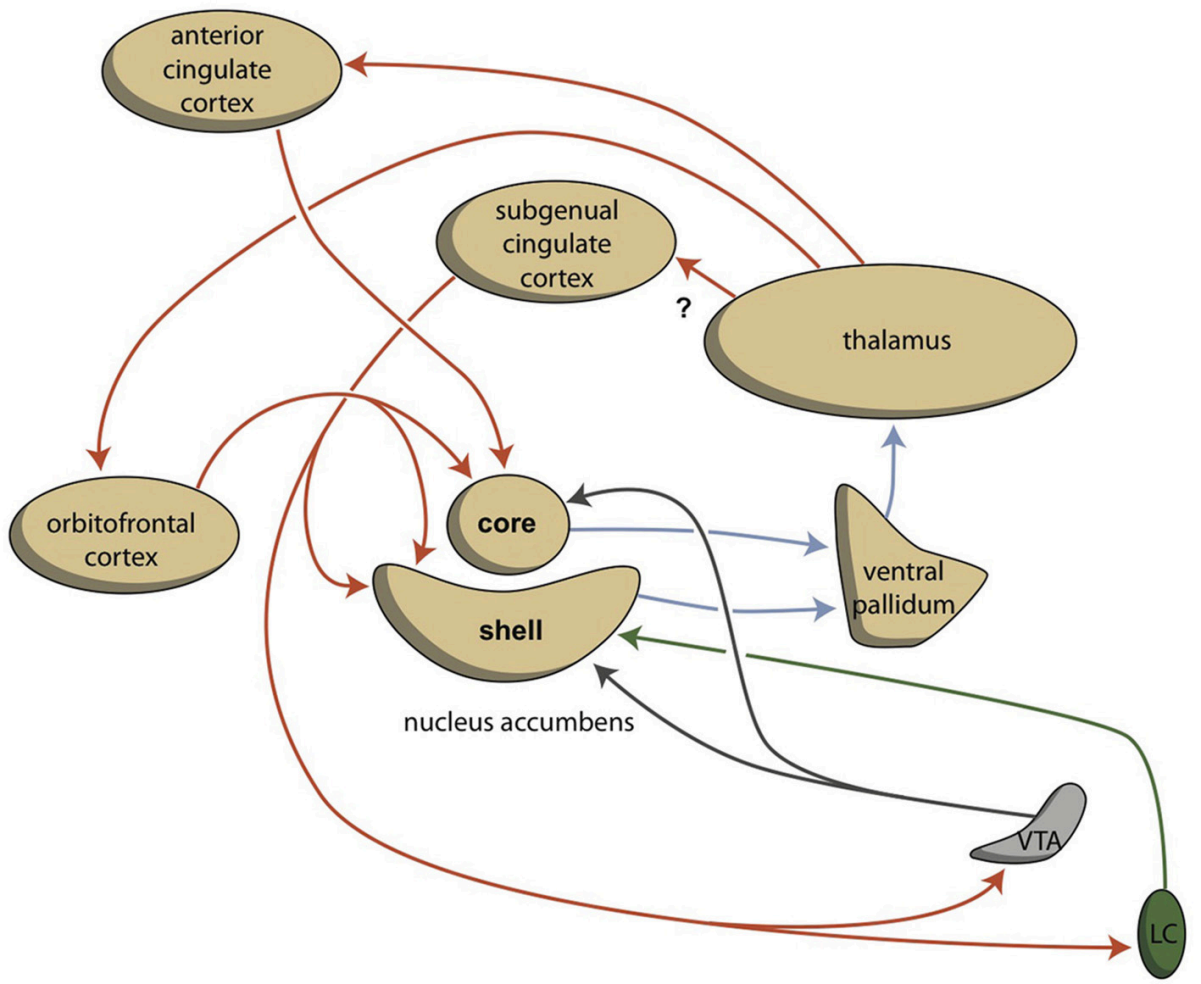
**Cortico-striatal-thalamo-cortical re-entry circuits including core and shell parts of the accumbens nucleus (adapted from Dalley et al., **2008**)**. The existence of the re-entry circuit including the subgenual cingulate cortex is still hypothetical. LC, locus coeruleus; VTA, ventral tegmental area. Red arrows = glutamatergic, blue arrows = GABAergic; green arrows = adrenergic; gray arrows = dopaminergic.

Behavior can be a reaction to an influence in the environment, or it can also be generated by the individual. To enable this reactive vs. proactive behavior, motivation comes into play (Rolls, 1999; Stuss and Knight, 2002). Three stages of behavioral motivation can be distinguished: general motivation, initiative, and selective precedence-conveying (via inhibition). The OFC plays a significant role in regulating these processes by delivering input to the ventral striatum, the anterior cingulate cortex, and the amygdala. Although the extrapyramidal and limbic circuits regulate two different types of behavior (cognitive and intuitive, respectively), the individual must be highly motivated to express these conducts. This motivation requires the involvement of two specific structures: the NAcbC and the NAcbS (Figure 3; Groenewegen, 2007; Dalley et al., 2008; Loonen and Stahl, 2011). The NAcbC motivates the individual toward behavior that may lead to a feeling of reward; while, the NAcbS motivates the individual toward behavior that may lead to escape from *misery* (Loonen and Ivanova, 2016b). When high stimulation of these motivations suddenly ceases, the individual experiences feelings of pleasure (NAcbC) or feelings of happiness (NAcbS). Therefore, we distinguish between circuits that regulate pleasure and those that regulate happiness (Loonen and Ivanova, 2015, 2016a,b). In clinical depression, both circuits seem to be dysfunctional. Low activity in the reward-seeking system results in the inability to experience pleasure (anhedonia), lack of energy, and indecisiveness. Particularly high activity in the *misery*-fleeing system results in continuous worrying, negative expectations, and dysphoric feelings.

**Figure 4:**
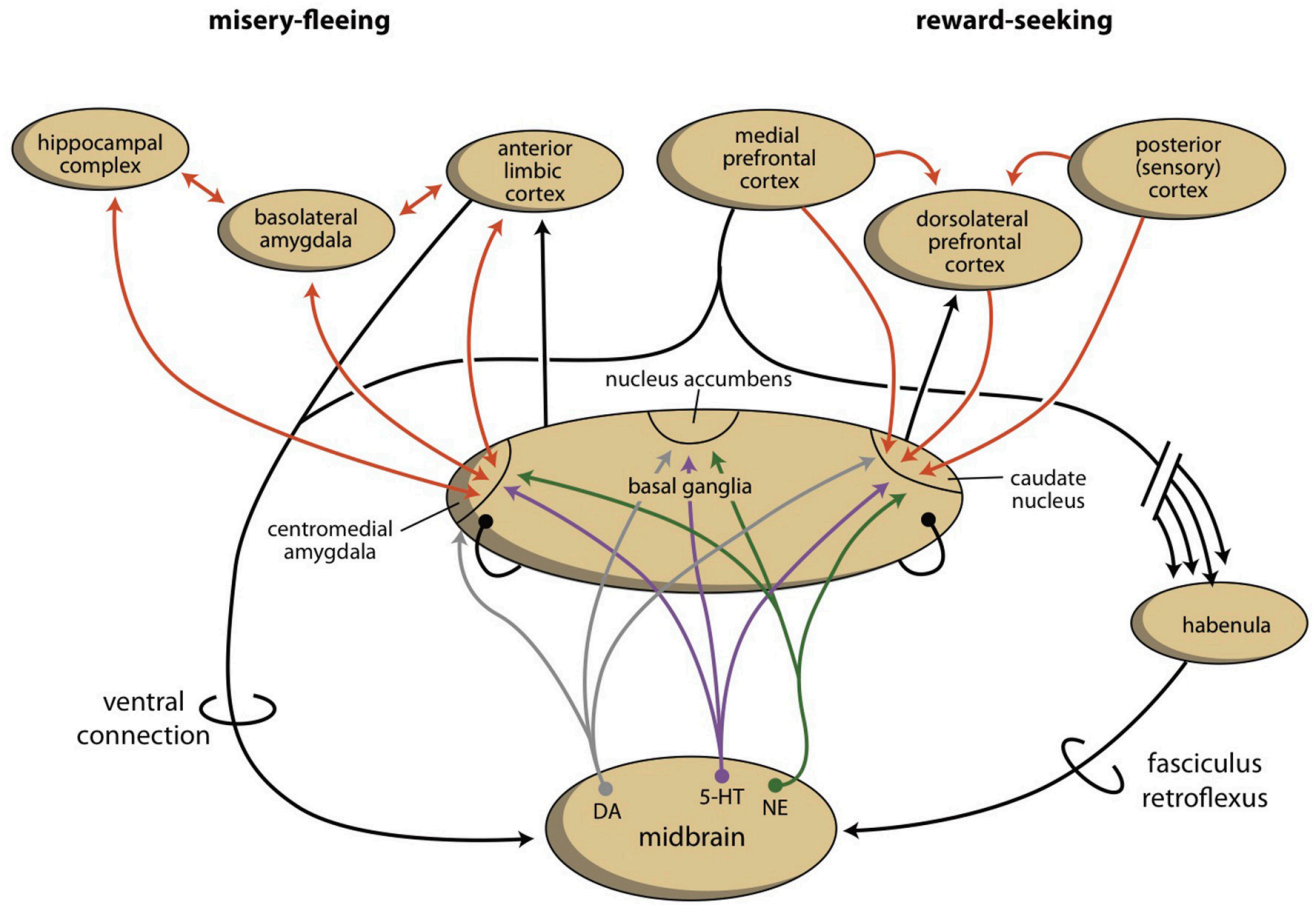
**Schematic representation of four levels of regulation of ***misery***-fleeing (left) and reward-seeking (right) behavior**.

The activities of the NAcbC and NAcbS, in turn, are regulated by monoaminergic nuclei within the midbrain. These nuclei transmit signals through dopaminergic (ventral tegmental area, VTA), adrenergic (norepinephrine, locus coeruleus), and serotonergic (raphe nuclei) tracts. In addition to their direct regulation of the NAcbC and/or NAcbS (Loonen and Ivanova, 2016b; Loonen et al., 2016), these monoaminergic nuclei regulate the activity of other, first relay-station basal ganglia and important parts of other areas in the forebrain. Therefore, behavioral output is controlled at three levels within the brain (Figure 4). The highest level is the cerebral cortex (isocortex, limbic cortex, corticoid amygdala, and hippocampal complex). The second level is the subcortical forebrain (dorsal striatum, ventral striatum, and extended amygdala). The third level is the midbrain (monoaminergic regulation centers).

As part of our depression model, we suggest that a fourth regulatory system exists, the habenula, which connects the cerebral cortex and midbrain systems (Figure 4; Loonen and Ivanova, 2016a,b; Loonen et al., 2016). Based on the regulation of appetitive behavior in lampreys, the lateral habenula (LHb) has an important regulatory function (Loonen and Ivanova, 2015). In the lamprey, when a behavior is particularly rewarding, the LHb promotes this behavior by intensifying stimulation of the phylogenetic homolog of the VTA. However, when the reward is smaller than expected or absent, the behavior is inhibited by affecting the VTA-equivalent in the lamprey. The medial habenula (MHb) appears to play a similar role with respect to *misery*-fleeing behavior (Robertson et al., 2014; Loonen and Ivanova, 2016c). The habenula belongs to the epithalamus, which also harbors the pineal gland and the stria medullaris. The habenula's projections to the midbrain were well-conserved during vertebrate evolution (Hikosaka, 2010; Loonen and Ivanova, 2016c; Loonen et al., 2016). Although its input structures are different in early vertebrates, compared with higher vertebrates, they include important inputs from the PFC as well as the septal areas and the amygdala. The input directly and indirectly connects the VTA to the cerebral cortex via a dorsal connection. Moreover, a direct connection between the PFC and the monoaminergic midbrain centers affects the activity of the VTA via a ventral connection (Loonen et al., 2016).

In conclusion, the extrapyramidal and limbic subcortical systems regulate cognitive (rational) and instinctive behaviors, respectively. The intensity of behavior that ultimately leads to reward is controlled by the cortico-striato-thalamo-cortical (CSTC) circuit that includes the NAcbC. The intensity of behavior that ultimately leads to safety is controlled by the CSTC circuit that includes the NAcbS. On the temporal side of the brain, the amygdala determines the appropriateness of flight, fight, or appetitive responses. Based on attentive salience, it initiates the proper emotional component of behavior. On the dorsal side of the brain, the caudate nucleus determines the suitability of the available repertoire of skilled behaviors and selects the proper motor response to achieve the intended goals. The motivation to express these behaviors is regulated by monoaminergic centers within the midbrain. In turn, these monoaminergic centers are regulated by the cerebral cortex through a dorsal connection that travels through the septal area and through the MHb and LHb; in addition, the monoaminergic centers are regulated by a direct ventral connection, which possibly travels through the medial forebrain bundle.

## Bipolar depression and mania

Although manifestations of mania are essential for delineating a bipolar disorder, according to the classification of Diagnostic and Statistical Manual of Mental Disorders, Fifth Edition (DSM-5; American Psychiatric Association, 2013), depression is the most frequent clinical presentation in both bipolar I and bipolar II disorders (Judd et al., 2002, 2003; Kupka et al., 2007). Moreover, many patients suffer from a mixed state with the co-occurrence of both manic and depressive symptoms (Cassidy et al., 2008). On the other hand, a relevant proportion of patients report recurrent manic episodes without ever having major depression (Cuellar et al., 2005; Angst and Grobler, 2015). The DSM-5, distinguishes MDD with mixed features from bipolar disorder (Vieta and Valentí, 2013), and this may complicate the diagnosis even more (Koukopoulos and Sani, 2014; Faedda et al., 2015). From a neuro-psycho-pharmacological perspective, the conclusion that manic and depressive symptoms apparently intermingle in both bipolar and unipolar mood disorders are a real challenge when trying to clarify their pathophysiology.

Mania is characterized by an elevated, expansive, or unusually irritable mood, as well as notably increased and persistent goal-directed activity. In addition, talkativeness, racing thoughts, decreased sleep, increased energy levels, and excessive involvement in pleasurable activities may be evident. These symptoms indicate increased activity of the circuit regulating reward-driven behavior and this is supported by experimental data (Wessa et al., 2014; Whitton et al., 2015). According to the DSM classification, MDD is probably a heterogeneous mixture of conditions and disorders, which allow extensive symptomatic diversity. However, a systematic review of studies did not provide conclusive evidence for the existence of depressive symptom dimensions or symptomatic subtypes (van Loo et al., 2012). There is empirical support for a melancholic subtype of depression that is qualitatively distinct from other forms of depression (Leventhal and Rehm, 2005). An important characteristic is the high association with anhedonia, i.e., impairment in the capacity to experience pleasure or to respond effectively to the anticipation of pleasure. In MDD, a reward processing dysfunction is the basis of at least a subtype of the depressive syndromes. It is unclear whether a bipolar depressive episode is symptomatically distinguishable from a unipolar major depressive episode. Although there is some inconsistency in the results from several studies, four symptoms appear to consistently differentiate groups: persons with unipolar depression have more anxiety, activity, and somatization, and less anhedonia compared with people with bipolar depression (Cuellar et al., 2005). Other characteristics differentiate between these groups to a far larger extent than symptomatology does (Bowden, 2005; Cuellar et al., 2005; Hirschfeld, 2014).

These findings, when integrated with our model of two separate cortical-subcortical circuits regulating reward-seeking and *misery*-fleeing behaviors, suggest that these circuits independently induce the two described components of bipolar mood disorder: activity and mood. The reward-seeking circuit appears to regulate the activity component; while, the *misery*-fleeing circuit controls the mood component (Wessa et al., 2014). We suggest that these two components play a mutually variable role in euphoric mania, dysphoric mania, mixed manic and depressive states, and bipolar depression. In general, in MDD, the increased activity of the *misery*-fleeing circuit is a dominant factor and this circuit also inhibits the activity of the reward-seeking circuit to a variable extent (Loonen and Ivanova, 2016b). This may explain why unipolar depression is characterized by more anxiety and somatization than bipolar depression (Cuellar et al., 2005).

## What about the switch?

**Figure 5:**
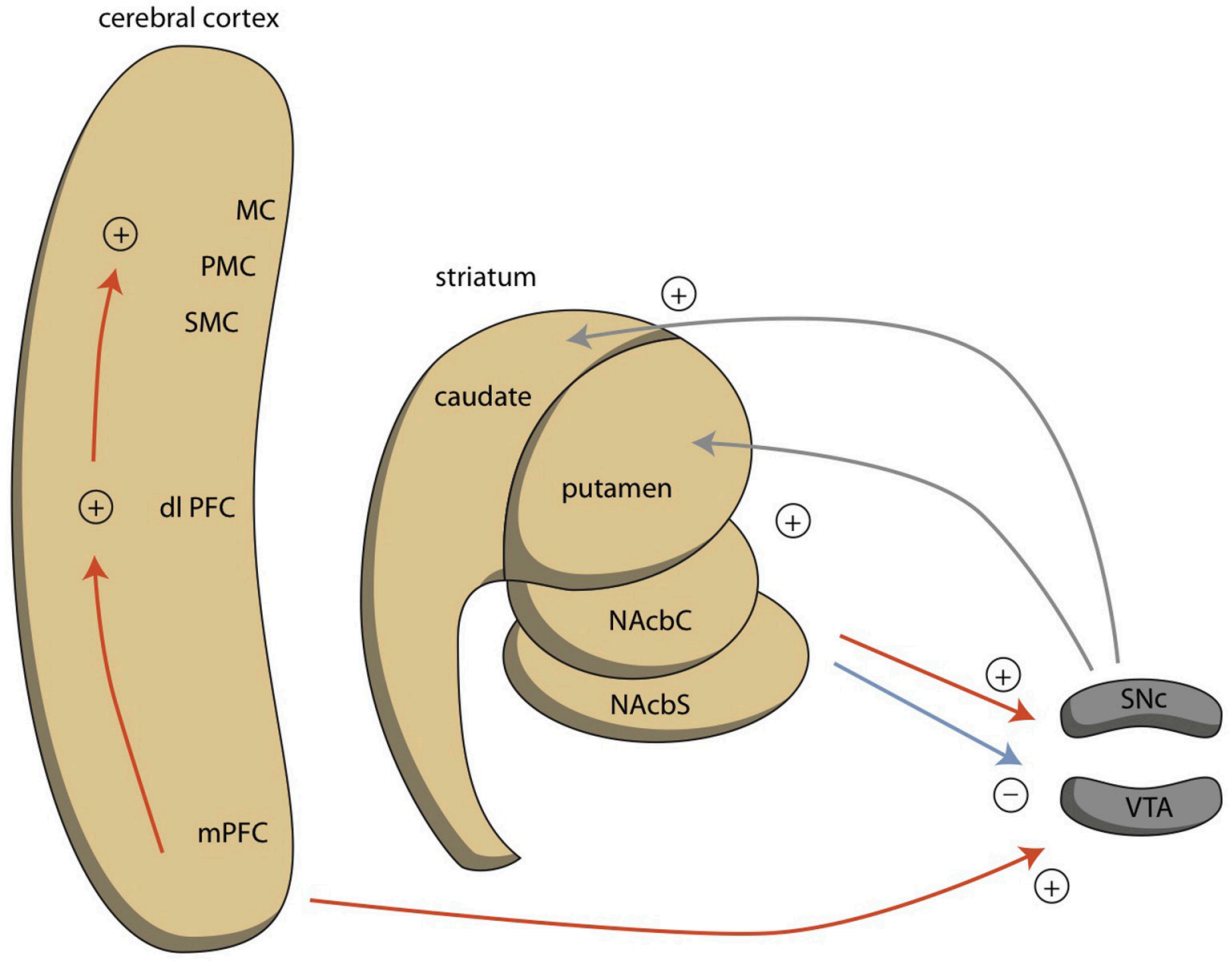
**Ventral connections regulating reward-seeking behavior**. dlPFC, dorsolateral prefrontal cortex; MC, motor cortex; mPFC, medial prefrontal cortex; NAcbC, core part of nucleus accumbens; NAcbS, shell part of nucleus accumbens; PMC, premotor cortex; SMC, supplementary motor cortex. Red = glutamatergic, blue = GABAergic; gray = dopaminergic.

In MDD, the dysfunction of the two circuits is probably caused by chronic hypermotivation to exhibit *misery*-fleeing behavior, which results in low activity of reward-seeking circuits (Loonen and Ivanova, 2016a,b). In bipolar disorder, another mechanism may be causing the dysregulation. Similar to the BAS dysregulation theory (Urošević et al., 2008; Nusslock et al., 2009; Bender and Alloy, 2011), we hypothesize that both increased and decreased motivation to exhibit goal-directed behavior is a specific driving force for the psychopathology in bipolar disorder. In mania, an excess level of motivation is observed; while, in depression, there is a reduction of motivation to exhibit goal-directed behavior. Reward-seeking behavior is regulated by the extrapyramidal cortical-subcortical circuit (Loonen et al., 2016) and the motivation to exhibit such behavior is regulated by the NAcbC. This nucleus also regulates the activity of more dorsal parts of the striatum, leading to increased pressure of thoughts (Figure 5). Within the NAcbC, increased dopaminergic activity leads to increased motivation.

We previously described that in the earliest vertebrate ancestors of humans—with a brain comparable to that of modern lampreys—the activity of the homolog of the VTA is regulated by the GPh within the parahippocampal lobe (Loonen and Ivanova, 2015). This structure regulates the activity of the LHb, which in turn can inhibit or disinhibit the homolog of the VTA by affecting the activity of the GABAergic rostromedial tegmental nucleus (RMTg). In the lamprey, the input of the GPh comes from the striatum, the lateral pallium (cortex), and as feedback from the homolog of the VTA (Stephenson-Jones et al., 2013). We suggested that during the evolution of these earliest vertebrates to humans, the forebrain of this primitive creature largely went up into the amygdaloid complex, next to the hippocampal complex and the oldest part of the limbic cortex (Loonen and Ivanova, 2015). Therefore, it is possible that the activity of the limbic human homolog of the GPh, is primarily regulated by the amygdaloid complex, next to (other) phylogenetically old cortical parts of the limbic system. This amygdaloid homolog of this GPh may be localized within the bed nucleus of the stria terminalis, which is the amygdaloid pallidum (Loonen and Ivanova, 2016c).

**Figure 6:**
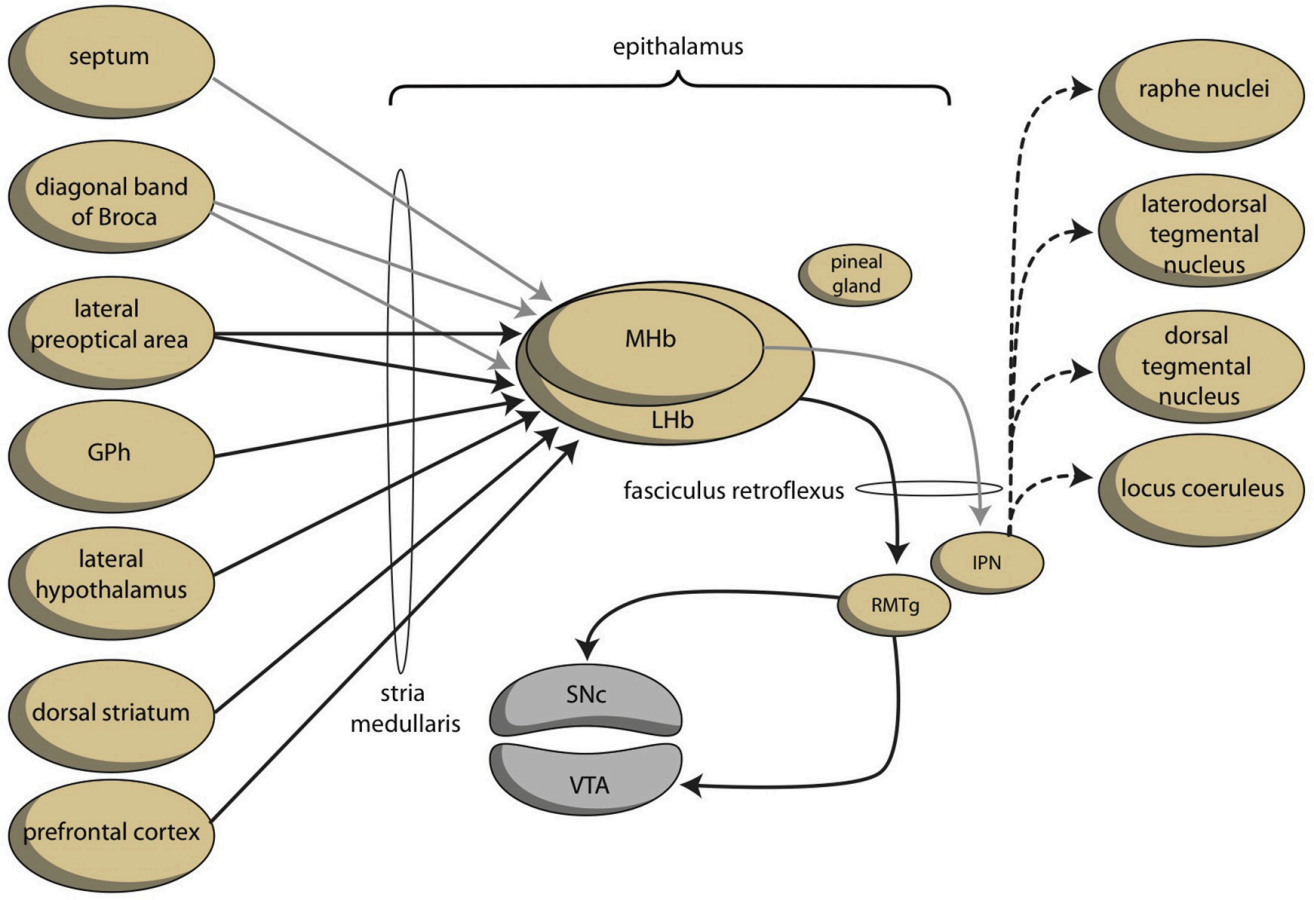
**Schematic representation of dorsal connections regulating reward-seeking and ***misery***-fleeing behaviors (adapted from Hikosaka, **2010**)**. The medial habenula is connected to and stimulates the lateral habenula. The color of the arrows was not determined neurochemically. GPh, putative habenula-projecting globus pallidus; IPN, interpeduncular nucleus; LHb, lateral habenula; MHb, medial habenula; RMTg, rostromedial tegmental nucleus; SNc, substantia nigra pars compacta; VTA, ventral tegmental area.

A core feature of bipolar disorder is the vulnerability to switch between under-activity (in depression) and over-activity (in mania) of the system regulating the motivation toward goal-directed behavior (Young and Dulcis, 2015). This switch can be induced by instability of the system regulating the activity of the mesencephalic dopaminergic system (VTA). This instability could, in turn, result from instability of the human homolog of the ancient GPh. This homolog of the GPh is expected to influence the activity of the LHb, which regulates the activity of the VTA. However, another possibility is that the circuitry of the habenula itself is unstable in bipolar disorder. The LHb is directly targeted by the MHb, but the MHb is not targeted by the LHb (Kim and Chang, 2005). The MHb is strongly connected to the cholinergic interpeduncular nucleus (IPN) within the midbrain (Figure 6). Elevated activity in the MHb and IPN have been linked to depression (Viswanath et al., 2014), and the MHb may also play an important role in anxiety and fear (Yamaguchi et al., 2013). Savitz and colleagues observed that bipolar, but not unipolar depressed, individuals have an abnormally reduced habenula volume in a high resolution MRI study (Savitz et al., 2011). Ranft et al. found a reduced volume of the MHb and LHb in the postmortem brains of depressed patients, compared with healthy controls and schizophrenic patients, and a reduced neuronal cell number of the MHb (Ranft et al., 2010). These authors studied both bipolar and unipolar depressed patients, but did not discriminate between them. A study in unipolar depressed patients did not find decreased total habenula volumes in patients with first-episode, long-lasting, recurrent or chronic depression (Carceller-Sindreu et al., 2015). Thus, although the evidence is still weak, structural changes within the habenula appear to be more evident in bipolar depression than in unipolar depression.

### Vulnerability to the pharmacological effects of antidepressants

A well-known phenomenon in the treatment of bipolar depression is that antidepressants may induce switching to a hypomanic or manic state (Salvadore et al., 2010; Young and Dulcis, 2015). Moreover, although definite evidence is lacking, chronic antidepressant treatment might also induce cycle acceleration (Carvalho et al., 2014). The risk of a switch appears to be less with serotonin reuptake inhibitors (Tondo et al., 2010) and when there is co-treatment with mood stabilizers (Licht et al., 2008). Tricyclic antidepressants inhibit both 5-hydroxytryptamine and norepinephrine (NE) uptake, and have antagonistic effects on a variety of neurotransmitter receptors (Richelson, 1994). Drug treatments resulting in elevated dopamine levels (levodopa, amphetamine) can induce mania-like states (Young and Dulcis, 2015). This might be related to increased stimulation of the nucleus accumbens. However, it is difficult to understand how chronic treatment with norepinephrine reuptake inhibitors can induce a mania-like state. It has become clear that downregulation of postsynaptic β-adrenoceptors is a consistent and robust effect of chronic treatment with most antidepressants, and this effect is accelerated by co-treatment with selective serotonin reuptake inhibitors (Anand and Charney, 2000). This downregulation would decrease the sensitivity of the NAcbS to stimulation by adrenergic terminals from the locus coeruleus complex (Loonen and Ivanova, 2015, 2016a). This decreased sensitivity to the induction of a stress response is not likely to result in a mania-like state.

The high affinity of tricyclic antidepressants to muscarinic receptors more likely explains the induced switching to (hypo)mania (Van Enkhuizen et al., 2015). Direct evidence for an anticholinergic drug-induced switch to (hypo)mania is missing, but anticholinergic drug-induced antidepressant effects are well-known (Hasselmann, 2014; Van Enkhuizen et al., 2015). Moreover, many findings in humans and animal studies indicate an association between cholinergic hyperactivity and bipolar depression (Van Enkhuizen et al., 2015). This appears to be related to stimulation of both muscarinic (particularly M2/M4) and nicotinic acetylcholine receptors (nAChRs; Van Enkhuizen et al., 2015). The MHb is widely known for its abundance of nAChRs (Viswanath et al., 2014). It is estimated that 90–100% of the neurons in the MHb express α3, α4, α5, β2, and/or β4 nAChR subunits (Sheffield et al., 2000) and this usually explains the involvement of the MHb–IPN pathway in critical aspects of nicotine dependence and nicotine-mediated behaviors (Antolin-Fontes et al., 2015). However, the nAChRs may also be involved in MHb–LHb connectivity and influence the activity of the dopaminergic VTA through the pathway from the LHb to the midbrain via the fasciculus retroflexus.

**Figure 7:**
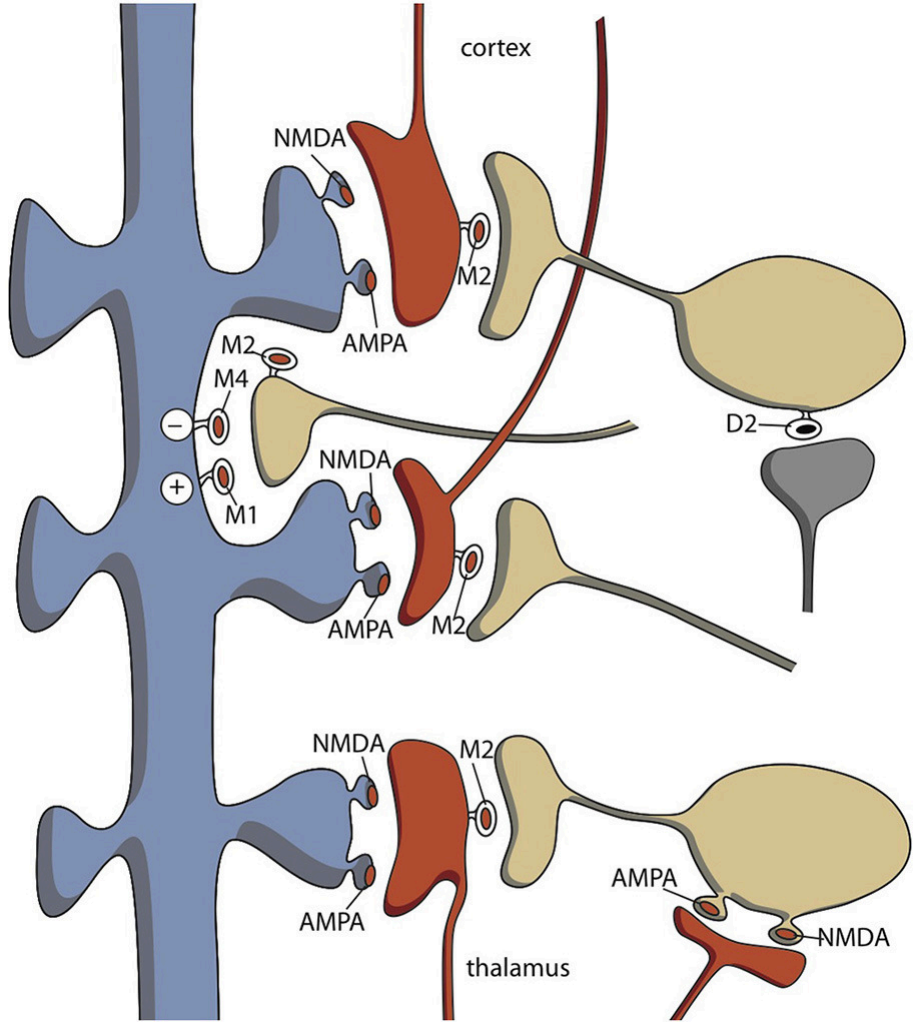
**Distribution of cholinergic neurons within the striatum**. Indirect pathway medium spiny neurons carry few M4-type muscarinic acetylcholine receptors. AMPA, α-amino-3-hydroxy-5-methyl-4-isoxazolepropionate glutamatergic receptors; D2, D2-type dopaminergic receptors; M1–M4, M1–M4 type muscarinic acetylcholine receptors; NMDA, N-Methyl-D-aspartate glutamatergic receptors. Red = glutamatergic terminals (corticostriatal and thalamostriatal); blue = GABAergic medium spiny neurons (unspecified direct or indirect pathway); yellow = cholinergic interneurons; gray = dopaminergic.

The functional antagonism between the cholinergic and dopaminergic systems is well-known, based on the treatment of parkinsonian side effects of dopamine antagonists. The effects of anticholinergics on parkinsonian side effects are attributed to the existence of cholinergic interneurons within the dorsal striatum. Medium spiny neurons (MSNs) constitute the vast majority of the striatal nerve cells and most of the remaining cells are one of three types of GABAergic interneurons (Groenewegen, 2003). However, although cholinergic interneurons make up only about 1% percent of the striatal neurons, these giant, aspiny neurons make the corpus striatum the largest cholinergic nucleus of the central nervous system (Calabresi et al., 2000; Pisani et al., 2007; Goldberg et al., 2012). These cholinergic interneurons show spontaneous activity. This basal activity is regulated by synaptic input. Acetylcholine stimulates both striatal nicotinic and muscarinic receptors. Muscarinic receptors are more widely spread and can be divided into M1-class (M1, M3, M5) and M2-class (M2, M4) receptors. The first class has stimulatory effects (vs. phospholipase C) and the second inhibitory (vs. adenylate cyclase). The cerebral distribution of acetylcholine muscarinic M1–M5 receptors was elucidated using immunological techniques (Levey et al., 1991; Hersch et al., 1994). Within the rat striatum, M1 and M4 receptors have a postsynaptic localization on the majority of striatal neurons and M2 receptors are typically localized on large (cholinergic) interneurons and act as inhibitory presynaptic autoreceptors (Figure 7). It may be concluded that the perikarya of MSNs carry M1 and M4; while, their dendritic spines carry M1, M3, and M4 receptors (Figure 7). Some MSNs must carry both M1 and M4 receptors. Santiago and Potter demonstrated that M4 receptors are primarily located on direct pathway MSNs. Only 14% of M4 receptors were found on indirect and 86% on direct pathway MSNs (Santiago and Potter, 2001). M1 receptors are expressed on almost all MSNs; while, M2 receptors are primary presynaptic autoreceptors, but are also present postsynaptically (Figure 7). Thus, by binding to M4 receptors, acetylcholine primarily inhibits MSNs of the direct pathway. This results in dominance of the indirect pathway, which has an inhibitory effect on behavioral output. This is similar to decreased dopaminergic activity and has classically been represented as a dopaminergic-cholinergic balance within the striatum (Van Enkhuizen et al., 2015). In summary, induction of a switch to (hypo)mania by tricyclic antidepressants is unlikely related to NE reuptake inhibition; rather, it is probably due to a blockade of M4 acetylcholine receptors. Cholinergic activation (e.g., by acetylcholinesterase inhibitors) may also induce depression by activation of nicotinic receptors, which activate MHb–LHb connections.

### Bipolar disorder and kindling (neuroplasticity/neurogenesis)

An influential conceptual framework to explain the impact of psychosocial stress on the switch to depression and/or (hypo)mania is the “kindling” hypothesis (Post, 1992). This hypothesis explains why unipolar and bipolar affective disorders are not only recurrent but also progressive in the sense that successive episodes occur with lesser stressors, after shorter intervals of remission, or with increased rapidity of cycling (Post, 1992). This hypothesis is described in detail elsewhere (Loonen and Ivanova, 2016a). Robert Post suggested that his theory was applicable to both unipolar depression and bipolar disorder. However, the evidence is far less conclusive in bipolar disorder than in MDD (Monroe and Harkness, 2005; Bender and Alloy, 2011). Studies on the kindling model of bipolar disorder have been inconsistent (Bender and Alloy, 2011). This is at least partly related to methodological difficulties in life stress measurements. However, another possibility is that the biological changes that accompany psychosocial stress-induced kindling (Post, 2007a; Loonen and Ivanova, 2016a), are primarily related to the vulnerability of the *misery*-fleeing circuit. This is especially true for the influence of the expression of brain-derived neurotrophic factor (BDNF; Post, 2007a,b). Sensitization of this *misery*-fleeing circuit could result in a pathological reaction to chronic and/or brief intense stress that could lead to an exaggerated response of this circuit. In MDD (unipolar depression), the reciprocal coupling between the *misery*-fleeing and reward-seeking circuits results in lower activity of the latter. In bipolar disorder, however, another pathogenic factor could have damaged the structure regulating the activity of the reward-seeking system. Hyperactivity of the *misery*-fleeing system can result in an aberrant response of the circuit regulating reward-seeking behavior, which could also lead to a switch to mania. This switch to mania or depression in bipolar disorder could be equivalent to the reaction that occurs when the mechanism regulating the reward-seeking circuit is challenged by similar psychosocial stress factors, which would cause a depressive episode in unipolar depression.

Such a mechanism could also explain the inconsistent findings concerning the relationship between (other) neurotrophins or immunological neurotrophic factors and bipolar disorder (Wu et al., 2014; Anderson and Maes, 2015; Réus et al., 2015; Muneer, 2016). The most widely investigated neurotrophic factor is BDNF (Wu et al., 2014), which was also investigated in MDD (Jentsch et al., 2015). This neurotrophic factor induces proliferation, survival, and differentiation of existing neurons, and the formation of new synapses. In MDD, there exists a clear relationship between BDNF levels and the depressive state, as well as the success of antidepressant therapy (Jentsch et al., 2015). However, the results in bipolar disorder are far more ambiguous (Wu et al., 2014). Studies aimed at other neurotrophins are relatively scarce (Wu et al., 2014). The putative neuroplastic effects of cytokines form the core of the neuro-immune hypothesis of MDD (Loonen and Ivanova, 2016a). Several findings suggest that inflammatory changes, demonstrated by increased levels of proinflammatory cytokines, decreased levels of anti-inflammatory cytokines, and activation of immune cells, are causally related to MDD (Jentsch et al., 2015; Réus et al., 2015). Also, in bipolar disorder, activation of microglia, which results in the production of cytokines, is believed to render the brain in a vulnerable and unstable state that precipitates mood disturbances (Réus et al., 2015; Muneer, 2016). Such changes are intimately linked to changes in oxidative and nitrosative stress and neuroregulatory tryptophan catabolites (Anderson and Maes, 2015; Réus et al., 2015; Muneer, 2016). It is unclear if these mechanisms result in neurotoxic changes within the connections to and from the LHb. The same is true with respect to neuroplastic changes induced by glucocorticoid steroids (Loonen and Ivanova, 2016a). The hypothalamic-pituitary-adrenal (HPA) axis is dysfunctional in both MDD and bipolar disorder (Muneer, 2016; Loonen and Ivanova, 2016a,b). Glucocorticoids are also capable of inducing mania (Young and Dulcis, 2015). Moreover, profound interactions between the HPA axis and the immune system exist and both endocrine and immune factors mediate neuroplastic effects (Loonen and Ivanova, 2016a). However, the exact pathogenic mechanism of how these interactions are linked to unipolar and bipolar mood disorders is far from clear.

Neuroplastic/neurotoxic changes that affect the functioning of the connection between the amygdaloid complex and the NAcbC, via the LHb and VTA, may explain the altered activity levels during the different affective episodes of bipolar disorder. There are numerous ways this system is influenced by exaggerated glutamatergic neurotransmission or decreased GABAergic neurotransmission due to neurotoxicity. In our opinion, the integrity of this “dorsal” connection in bipolar disorder should be the major subject of future investigations.

### Bipolar disorder and biorhythms

The habenular nuclei are paired structures located in the dorsomedial posterior thalamus and are closely associated with the pineal gland, which it has reciprocal connections with (Hikosaka, 2010; Benarroch, 2015). Both the MHb and LHb receive their inputs via the stria medullaris and, together with the pineal gland, these structures constitute the epithalamus (Figure 6). The pineal gland (epiphysis) produces melatonin during the dark period of the day-night cycle in both diurnal and nocturnal vertebrates (Macchi and Bruce, 2004). The close association of the habenula with the pineal gland may explain the role of the habenula in the control of sleep and circadian fluctuation of psychomotor activity. This last phenomenon is probably part of the role of the habenula in an extensive set of cyclical changes in physiology and behavior. These cyclical processes are often synchronized to the changing environmental light-dark cycle. The circadian rhythms are controlled by an endogenous clock, located in the suprachiasmatic nuclei of the hypothalamus, and are dependent on an oscillatory expression of certain clock genes (McCarthy and Welsh, 2012). Connections with the retina are supposed to determine the sensitivity to changes between day-light and night-darkness (Macchi and Bruce, 2004; McCarthy and Welsh, 2012). These suprachiasmatic nuclei are directly connected with the habenula and possibly also via the paraventricular nucleus of the hypothalamus (Salaberry and Mendoza, 2016). The firing of LHb neurons shows circadian rhythmicity (Guilding and Piggins, 2007), even in slice preparations (Zhao and Rusak, 2005; Hikosaka, 2010); although, the physiological relevance of this can be disputed. Guilding et al. established that neurons and non-neuronal cells in the epithalamus may have a role as circadian oscillators in the temporal regulation of processes and behavior (Guilding et al., 2010). Moreover, many animals can detect changes in electric or magnetic fields and use this information to perform various behaviors, such as navigating and prey-capture. In the lamprey, this electromagnetic-induced behavior seems to be controlled by the MHb–IPN system (Chung-Davidson et al., 2004; Hikosaka, 2010). These properties may be related to the role of the habenula in the annual migration of, e.g., birds.

Disturbance of the circadian timing system may play a fundamental role in the etiology of bipolar disorder (Gonzalez, 2014). This probably explains the susceptibility of patients with bipolar disorder for disruption of biological rhythms (Souêtre et al., 1989; Gonzalez, 2014; Young and Dulcis, 2015). The relationship of the regulation of the circadian rhythm with the sleep-wake cycles may be linked with the mood swings in bipolar disorder. Sleep disturbance is a hallmark of bipolar disorder and numerous studies illustrate the relation between sleep quality and affective symptoms (Gonzalez, 2014). Sleep deprivation has antidepressant effects and also may precede a manic episode. Moreover, at least a subset of bipolar patients showed a seasonal pattern in their mood fluctuations (Gonzalez, 2014). There exists some indirect evidence that the social, behavioral, and physiological rhythm disturbances in bipolar disorder might be related to dysfunctional clock genes (Roybal et al., 2007; Gonzalez, 2014). The studied clock gene *CLOCK* (Circadian Locomotor Output Cycles Kaput) and its binding partner BMAL1 (also known as Aryl hydrocarbon receptor nuclear translocator-like protein 1 or ARNTL1) were associated with bipolar disorder phenomena in genetic studies (Roybal et al., 2007; McCarthy and Welsh, 2012; Young and Dulcis, 2015). However, to the best of our knowledge, the genes coding for CLOCK/BMAL 1 have not (yet) been associated with bipolar disorder in genome-wide association studies (Craddock and Sklar, 2013). An interesting idea was published in 2012 that indirectly linked bipolar disorder to a function of the habenula. A hypothesis of the evolutionary origin of bipolar disorder was that bipolar behavior evolved from human adaptations to the selective pressures of severe climatic conditions in the northern temperate zone, such as during the Pleistocene (Sherman, 2012). This author suggested that modern day humans who first appeared near the equator in Africa acquired these genes by mating with Neanderthals, who are considered the primary source of these genes (Sherman, 2012; Young and Dulcis, 2015). This is related to ideas about the acquisition of important aspects of our immune system (Ségurel and Quintana-Murci, 2014). Recent work in humans shows that this can be achieved by acquiring novel selected alleles through admixture with extinct hominins such as Neanderthals or Denisovans (Ségurel and Quintana-Murci, 2014). Adaptation to changing climatic conditions is also the main driving force to initiate and maintain animal migration and, although direct evidence is missing, the habenula is a good candidate to play an important role in enabling this behavior by stimulating the motivation to get and keep on moving.

### Bipolar disorder and genetics

A thorough description of the genetics of bipolar disorder is not suitable here (Craddock and Sklar, 2013). We only want to refer to the results of genome-wide association studies, which until now have failed to identify a clear set of genes that dominantly contribute to the chance of having bipolar disorder. In contrast, strong evidence exists for a polygenic contribution to the risk for bipolar disorder. This is irrespective of the findings from an approach that found a genetic association for certain clock genes (McCarthy and Welsh, 2012; Young and Dulcis, 2015) or factors associated with a response to mood stabilizers, like lithium (Rybakowski, 2014a,b).

## Conclusions and future research

The classification of mood disorders in the DSM-IV-TR and DSM-5 does little to clarify the differences in the biological background of bipolar and unipolar disorders. We hypothesize that in the most prevalent form of depression, chronic, or intense exposure to psychological stress factors or other biological reactions lead to dysfunctional hyperactivity of the *misery*-fleeing regulatory circuit (resulting in dysphoria, continuous worrying, and negative expectations in humans). Hyperactivity of this first circuit leads to hypoactivity of the reward-seeking regulatory circuit (resulting in lack of pleasure, low energy, and indecisiveness) as the two are reciprocally coupled (Loonen and Ivanova, 2016a,b). In a subset of patients with mood disorders, the response of the circuit regulating reward-seeking (goal-directed) behavior is variable due to a separate pathological mechanism. This is probably caused by damage to the subcortical mechanism regulating the activity of these reward-seeking (extrapyramidal) circuits. Altered activity of these circuits may lead to general behavioral hypoactivity (depression), partly mismatching hyperactivity (agitated depression, dysphoric mania), or full behavioral hyperactivity (hypomania, mania). In our opinion, this instability of subcortical mechanisms regulating the reward-seeking extrapyramidal circuits forms the pathogenic core of bipolar disorder, and this may differ from ordinary unipolar depression. Altered activity of the cortical-subcortical circuits may alter the frontal cortical functioning, but not the other way around as was indirectly suggested in the BAS dysregulation theory (Urošević et al., 2008). Moreover, the BIS may represent another activity of the same reward-seeking system with the feeling of punishment being represented by hyperactivation of the *misery*-fleeing system. In our opinion, this subcortical control does not affect how the frontal cerebral cortex influences the subcortical structures (Nusslock et al., 2009). Results from older studies of biological factors (e.g., genetic, environmental, neuroplastic, neurotoxic) related to bipolar mood swings should be re-interpreted, when possible, and should specifically address the vulnerability of the reward-seeking circuit to aberrant reactions resulting from stimulation of the circuits regulating *misery*-fleeing behavior.

**Figure 8:**
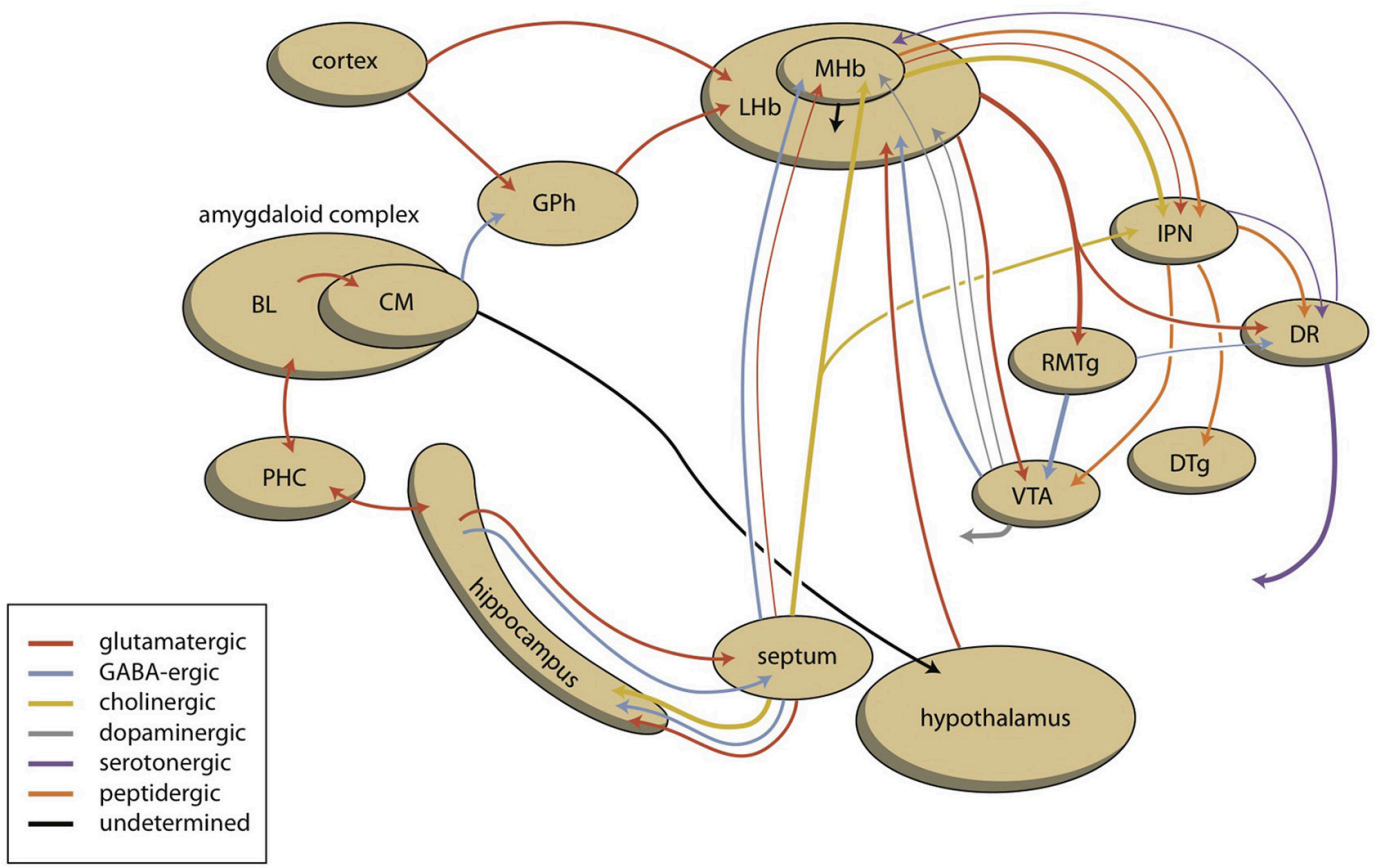
**Scheme showing the dorsal connectivity of the limbic (cortical) system to the midbrain (the upper left part of the scheme varies pro system)**. BL, basolateral (cortical) part; CM, centromedial (nuclear) part; DR, dorsal raphe nucleus; DTg, dorsal tegmental nucleus; GPh, limbic part of the habenula-projecting globus pallidus; IPD, interpeduncular nucleus; LHb, lateral habenula; MHb, medial habenula; PHC, parahippocampal cortex; RMTg, rostromedial tegmental nucleus; VTA, ventral tegmental area.

It is unclear if the pathway making the connections between the amygdaloid complex and the GPh, LHb, RMTg, VTA, and finally the NAcbC (Figure 8) is primary affected in such bipolar disorder or if the regulation of the LHb by the MHb is aberrant. Many findings indicate the involvement of the habenula (biorhythm and seasonal aspects of bipolar disorder, the results of neuroimaging studies, pharmacological findings). However, there is currently not a clear model of how the bipolar mood switch occurs and how the reward-seeking circuit and habenula are involved. In our opinion, the development and testing of such a model is one of the biggest challenges of future bipolar disorder research.

## Author contributions

AL developed the ideas and wrote the draft manuscript in collaboration with SI. RK commented on these ideas as an expert on the clinical and neurobiological background of bipolar disorder.

### Conflict of interest statement

The authors declare that the research was conducted in the absence of any commercial or financial relationships that could be construed as a potential conflict of interest.

## Abbreviations

BAS: behavioral approach system
BDNF: brain-derived neurotrophic factor
BIS: behavioral inhibition system
CSTC: cortico-striato-thalamo-cortical
DSM: diagnostic and statistical manual of mental disorders
GABA: gamma aminobutyric acid
GPh: habenula-projecting globus pallidus
HPA: hypothalamic-pituitary-adrenal
IPN: interpeduncular nucleus
LHb: lateral habenula
LTP: long term potentiation
LTD: long term depression
MHb: medial habenula
MDD: major depressive disorder
MSN: medium spiny neuron
NAcbC: nucleus accumbens core
NAcbS: nucleus accumbens shell
nAChRs: nicotinic acetylcholine receptors
NE: norepinephrine
OFC: orbitofrontal cortex
PFC: prefrontal cortex
RMTg: rostromedial tegmental nucleus
VTA: ventral tegmental area.
